# The Prevalence of Various Autoimmune Comorbidities in Patients with Inflammatory Bowel Disease

**DOI:** 10.3390/epidemiologia6030052

**Published:** 2025-09-02

**Authors:** Bipneet Singh, Shivam Kalra, Tejasvini Khanna, Isha Kohli, Vikash Kumar, Aalam Sohal, Divyesh Sejpal

**Affiliations:** 1 Henry Ford Jackson, 159, W Michigan Ave, Jackson, MI 49201, USA; 2 Trident Medical Center, North Charleston, SC 29406, USA; drshivamkalra@gmail.com; 3 Department of Medicine, Maulana Azad Medical College, New Delhi 110002, India; tkhanna111298@gmail.com; 4 Graduate School of Public Health, Icahn School of Medicine at Mount Sinai, New York, NY 10029, USA; ishakohli689@gmail.com; 5 Department of Gastroenterology, Creighton University, Phoenix, AZ 85012, USA; kumarvikashmd@gmail.com (V.K.); aalamsohal@gmail.com (A.S.); 6 Department of Gastroenterology and Hepatology, University of Texas Medical Branch, Galveston, TX 77555, USA

**Keywords:** inflammatory bowel disease, rheumatology, autoimmunity, psoriasis, polymyositis

## Abstract

**Introduction:** Patients with inflammatory bowel disease (IBD) are at increased risk of developing other autoimmune disorders due to possible shared genetic, environmental, and immunological mechanisms. While autoimmune diseases are frequently observed in patients with IBD, data quantifying their inpatient prevalence and their association with outcomes such as mortality remain limited. **Methods:** National Inpatient Sample (NIS) 2016–2020 and International Classification of Diseases 10th Version, Clinical Modification (ICD-10-CM) diagnosis codes were used to identify patients with IBD and autoimmune conditions. A multivariate logistic regression analysis to identify an association between various autoimmune diseases and various IBDs was performed. **Results:** The study population included 141,478,025 patients. An association was found between 24 autoimmune conditions and IBD. **Conclusions:** Our study identified autoimmune comorbidities that are more prevalent in IBD patients. We found that polymyositis, AIHA, ITP, and thrombotic microangiopathy are associated with a higher risk of in-hospital mortality. Psoriasis and hypothyroidism are associated with a lower risk of in-hospital mortality. Further studies are needed to explore the mechanisms responsible.

## 1. Introduction

Inflammatory bowel disease (IBD) comprises Crohn’s disease (CD) and Ulcerative Colitis (UC) The etiopathogenesis of IBD is not completely known, but it has been suggested that an abnormal intestinal immune response to intestinal flora results in repetitive episodes of gut inflammation [1]. Other factors reported to play a role include environmental triggers and genetic predisposition [2]. Similar triggers and genetic predisposition have also been reported to exist in other autoimmune diseases such as rheumatoid arthritis (RA), ankylosing spondylitis, type 1 diabetes, and celiac disease [3]. Genome-wide association studies have reported similar genetic susceptibility loci in both IBD and psoriasis [4]. RA, a TH-1 driven disease, has been shown to have a shared susceptibility locus IRF5, deoxyribonucleic acid (DNA) polymorphisms, and a decrease in the gut microbiota of Bacteroides similar to IBD [5,6,7,8]. Similar genetic susceptibility loci have also been noted between IBD and other autoimmune diseases.

Studies have pointed towards a role of the gut microbiome in the development of IBD [9,10,11]. The gut microbiota of IBD patients is less diverse, with fewer bacteria and a different microbial metabolite profile as compared to healthy people [12]. Additionally, the use of antibiotics in patients with IBD has been associated with an increased risk of developing autoimmune disease. Aside from the extraintestinal manifestations reported in IBD, multiple population-based studies have reported an association between IBD and other autoimmune conditions such as psoriasis, multiple sclerosis, and vasculitis. Increased disease severity in IBD has been associated with a higher risk of developing other autoimmune diseases [9,13,14,15,16,17]. The use of amino salicylates has been associated with a reduced risk of developing other autoimmune diseases. In this study, we aim to use nationally available United States (US) inpatient data to quantify the prevalence of 24 autoimmune conditions in hospitalized patients with IBD and to evaluate their association with in-hospital mortality.

## 2. Methods

### 2.1. Data Source

The National Inpatient Sample (NIS) is the largest database of inpatient hospital stays and is maintained by the Healthcare Cost and Utilization Project [18]. Using information from a 20% stratified sample of hospitals in the United States from 37 states, it reliably estimates the patient population and outcomes. Every hospitalization is de-identified and is kept as a unique entry in the NIS. IRB approval was not required since the data was de-identified, and publicly available.

### 2.2. Study Population

**Figure 1:**
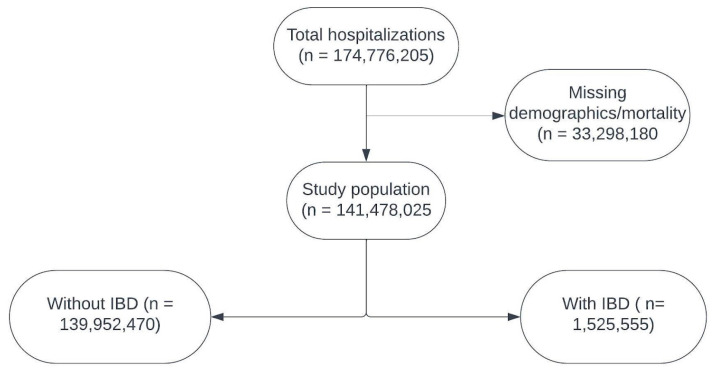
Population included in the study.

Our study included all the adult patients who were admitted between 2016–2020. Patients with missing demographics were excluded. The International Classification of Diseases 10th Version, Clinical Modification (ICD-10-CM) diagnosis codes were used to identify patients with IBD. The patients were stratified into two groups—those with IBD (both UC and CD) and those without IBD. A different analysis was not performed for UC and CD separately. The inclusion is shown in Figure 1.

### 2.3. Study Variables

Information on patient demographics, such as age, gender, race, primary insurance, and median income quartile, was collected. Further information on common autoimmune conditions was also obtained. These included autoimmune cutaneous disorders (pemphigus vulgaris (PV), bullous pemphigoid (BP), psoriasis), autoimmune muscular disorders (polymyositis, myasthenia gravis (MG)), autoimmune hematological conditions (autoimmune hemolytic anemia (AIHA), idiopathic thrombocytopenic purpura (ITP)), celiac disease (CD), autoimmune neurologic disorders (amyotrophic lateral sclerosis (ALS), multiple sclerosis (MS), Guillain-Barre syndrome (GBS)), autoimmune endocrine disorders (Addison’s disease (AD), hypothyroidism, Graves’ disease (GD) and type 1 diabetes mellitus (DM)), autoimmune vasculitis (Takayasu arteritis, thrombotic microangiopathy, polyarteritis nodosa (PAN), giant cell arteritis (GCA), Wegner’s granulomatosis (WG)), autoimmune liver diseases (primary biliary cholangitis (PBC), primary sclerosing cholangitis (PSC) and autoimmune hepatitis (AIH)) and autoimmune connective tissue disorders (ankylosing spondylitis, Bechet’s disease, Rheumatoid arthritis, Sarcoidosis, Sjogren syndrome, Systemic lupus erythematosus (SLE), polymyalgia rheumatica (PR) and discoid lupus erythematosus (DLE). The ICD-10 codes were used to identify these diagnoses. In-hospital mortality, length of stay (LOS), and total hospitalization charges were also checked.

### 2.4. Statistical Analysis

We used hospital-level discharge weights to generate national estimates. To compare categorical variables, the Chi-square test was used, while an independent sample *t*-test was used for continuous variables. A multivariate logistic regression analysis to identify an association between various autoimmune diseases and various IBDs was performed. A check for multicollinearity among independent variables was not conducted. Adjustments for patient demographics and the Elixhauser comorbidity index, a well-validated index based on ICD 10-CM codes meant to be used in large administrative data to reliably estimate the comorbidity burden, were done [19].

The relationship between various autoimmune disorders and in-hospital mortality was assessed among patients with IBD using a multivariate logistic regression model. Only the variables that were noted to be less than 0.1 on univariate analysis were included. Besides the autoimmune disorders, other factors that were selected in the multivariate analysis were patient demographics. The adjusted odds ratios were calculated with a 95% confidence interval. A type I error of 0.05 was statistically significant. We used STATA 17.0 (Texas) to analyze the data.

## 3. Results

### 3.1. Patient Demographics

The study population included 141,478,025 patients. Of these, 1,525,555 (1.1%) had IBD. Compared to patients without IBD, those with IBD were more likely to be aged 18–44 years (34.0% vs. 28%, *p* < 0.001), Males (44% vs. 42.5%, *p* < 0.001), Whites (79.0% vs. 67%, *p* < 0.001) and those with private insurance (36% vs. 27%, *p* < 0.001) in the IBD group compared to those without. The results are presented in Table 1.

### 3.2. Autoimmune Comorbidities and IBD

#### 3.2.1. Autoimmune Cutaneous Disorders

There were no significant differences in the rates of bullous pemphigoid and pemphigus vulgaris between patients with IBD and those without IBD. A higher incidence of psoriasis was noted in patients with IBD (1.3% vs. 0.5%, *p* < 0.001). After adjusting for confounding factors, patients with IBD had 2.35 times higher odds of having psoriasis (aOR-2.35, 95% CI-2.38–2.43, *p* < 0.001). The results of an association between autoimmune comorbidities and IBD are presented in Table 2.

#### 3.2.2. Autoimmune Muscular Disorders

There were no significant differences in the rates of polymyositis and myasthenia gravis between patients in the IBD group and the non-IBD group.

#### 3.2.3. Autoimmune Hematological Disorders

Patients with IBD had a higher incidence of autoimmune AIHA, ITP and pernicious anemia than those without. After adjusting for confounding factors, IBD was associated with a 30%, 10% and 147% higher odds of developing AIHA (*p* = 0.001), ITP (*p* = 0.01), and pernicious anemia (*p* < 0.001), respectively.

#### 3.2.4. Autoimmune Gastrointestinal and Hepatic Disorders

There was a higher incidence of celiac disease, PBC, AIH, and PSC in patients with IBD than in those without. After adjusting for confounding factors, patients with IBD had 208%, 282 and 301% higher odds of developing celiac disease (*p* < 0.001), PBC (*p* < 0.001), and AIH (*p* < 0.001). Patients with IBD were also noted to have 80 times higher odds of developing PSC (*p* < 0.001).

#### 3.2.5. Autoimmune Neurologic Disorders

There were no significant differences (*p* > 0.05) in the rates of amyotrophic lateral sclerosis between patients with or without. IBD: A higher incidence of multiple sclerosis and GBS was noted in patients with IBD, but after adjusting for confounding factors, IBD was not associated with multiple sclerosis and GBS (*p* > 0.05).

#### 3.2.6. Autoimmune Endocrine Disorders

Patients with IBD had a higher incidence of Addison’s disease (0.3% vs. 0.01%, *p* < 0.001), Grave’s disease (0.2% vs. 0.1%, *p* < 0.001) and hypothyroidism. Patients with IBD had a lower incidence of type 1 DM (1% vs. 1.2%, *p* < 0.001). After adjusting for confounding factors, patients with IBD had 168% and 140% higher odds of Addison’s disease and Grave’s disease than those without. Patients with IBD had 48% lower odds of developing type 1 DM.

#### 3.2.7. Autoimmune Connective Tissue Disorders

Patients with IBD had a higher incidence of ankylosing spondylitis (0.5% vs. 0.06%, *p* < 0.001), Bechet’s disease (0.05% vs. 0.01%, *p* < 0.001), RA (4% vs. 2%, *p* < 0.001), Sjogren’s syndrome (0.3% vs. 0.2%, *p* < 0.001), SLE (1% vs. 0.6%, *p* < 0.001), and DLE (0.07% vs. 0.04%, *p* < 0.001). The highest association among autoimmune connective disorders was noted between IBD and ankylosing spondylitis (aOR-8.88, 95% CI-8.43–9.38, *p* < 0.001). A positive association, after adjusting for confounding factors, was also noted, as mentioned in Table 2.

#### 3.2.8. Autoimmune Vasculitis

Patients with IBD had a higher incidence of Takayasu arteritis, PAN, GCA, and Wegener’s granulomatosis than those without. The strongest association was noted between IBD and Takayasu arteritis (aOR-3.15, 95% CI-2.28–4.34, *p* < 0.001) after adjusting for confounding factors. Other conditions noted to have a positive association included PAN, GCA, and Wegener’s granulomatosis.

Strongest associations were observed with primary sclerosing cholangitis, ankylosing spondylitis, autoimmune hepatitis, and celiac disease. Notably, IBD was also independently associated with several less commonly reported conditions such as Addison’s disease, Bechet’s disease, and Takayasu arteritis. A few autoimmune disorders showed no significant association or even an inverse relationship (e.g., type 1 diabetes), the overall pattern hinted at a broader systemic immune dysregulation in IBD.

### 3.3. Factors Associated with In-Hospital Mortality Among Patients with IBD

Odds of in-hospital mortality were found to be elevated in age 45–65 (aOR 2.47, *p* < 0.001) and >65 (aOR 5.05, *p* < 0.001). Female patients were found to have 15% lower odds of mortality compared to males (aOR 0.85, *p* < 0.001). The autoimmune comorbidities associated with lower odds of in-hospital mortality among IBD patients were psoriasis vulgaris (aOR 0.53, *p* < 0.001) and hypothyroidism (aOR 0.70, *p* < 0.001). The autoimmune conditions associated with increased odds of in-hospital mortality were polymyositis (aOR 2.56, *p* = 0.017), AIHA (aOR 2.60, *p* = 0.006), ITP (aOR 2.19, *p* < 0.001), and thrombotic microangiopathy (aOR 4.94, *p* < 0.001). A complete list of factors associated with mortality is presented in Table 3.

## 4. Discussion

Our study is one of the largest studies using the NIS to assess the relationship between IBD and other autoimmune conditions. Out of the 34 autoimmune conditions studied, an association was noted between IBD and 24 other autoimmune conditions.

### 4.1. Autoimmune Cutaneous Disorders

Among autoimmune cutaneous disorders, a strong positive association was noted between IBD and psoriasis. This association was documented as early as 1968 and has since been reproduced in various studies [20]. A recent cross-sectional study in North Carolina, a nationwide study in Canada, and a small study at the Mayo Clinic concur with these findings [9,14,21,22]. A pathogenesis involving IL 23R, IL 12B, CDKAL1, and PTPN22 is common to both IBD and psoriasis [23]. Anti-TNF therapy has paradoxically been implicated in inducing psoriatic lesions in IBD patients [20,23]. A meta-analysis has accordingly found a higher prevalence of psoriasis in IBD patients on anti-TNF therapy [24]. No association between IBD and other autoimmune skin disorders, namely bullous pemphigoid and pemphigus vulgaris, was found. Our results are discordant with the findings of a study using the Taiwanese national database that reported an association between bullous pemphigoid and IBD and a study conducted at the University of Toronto, which noted a higher prevalence of IBD in patients with pemphigus vulgaris [10,25]. Hence, further studies examining the association between IBD and autoimmune cutaneous disorders are needed.

### 4.2. Autoimmune Muscular Disorders

Our study did not find any association between polymyositis and IBD. Our results are not in agreement with previous studies that have found common genetic factors and a higher incidence of MS in both UC and CD [26]. Prior studies often relied on outpatient cohorts, which might indicate that such complications might not lead to inpatient admission, or smaller sample sizes, which may account for differing conclusions. Patients with IBD often report muscle weakness, and it is pertinent to differentiate polymyositis from steroid-induced myopathy via muscle biopsy [26]. The presence of polymyositis was associated with 2.5 times higher odds of in-hospital mortality among patients with IBD. MG has also been described in a handful of patients with IBD. Interestingly, thymectomy in patients with MG and IBD led to worsening in IBD symptoms as well, suggesting of a thymic role in IBD [27]. However, no association was noted in our study.

### 4.3. Autoimmune Hematological Disorders

A strong association was noted between IBD and autoimmune hematological disorders.

ITP has been described in UC patients in several case reports previously and attributed to alterations in immunoregulation and secondary immuno-stimulation from antigens in the gut lumen [13,28]. Previous literature reviews and multicenter studies support the association between IBD and AIHA [29]. The exact mechanism underlying this link is poorly understood. There is no compelling evidence to suggest that biologic therapy is responsible for AIHA. In some cases, IBD surgery has led to the resolution of AIHA, suggesting that the colon is the site of production or induction of anti-RBC antibodies [29]. An association between IBD and pernicious anemia has been detailed previously [30]. This is relevant since B12 deficiency is generally attributed to poor absorption in the inflamed ileum or in the setting of therapeutic resection; however, the possibility of concurrent pernicious anemia warrants consideration [30]. Screening for AIHA and ITP in patients with hematological disturbances, as the presence of these was noted to be associated with a higher risk of mortality in our study, is recommended.

### 4.4. Autoimmune Gastrointestinal and Hepatic Disorders

The association between IBD and autoimmune liver disease and celiac disease is well studied and has also been reported in our study [21,31]. The epidemiology and pathogenesis of celiac disease overlap with IBD, both are inflammatory diseases largely seen in young women and involve a Th1 response, IL-18 receptor dysfunction, and altered microbiota [31]. A study conducted in the Danish population reported PSC to be associated with both UC and CD, while AIH and PBC were noted to be higher only in UC patients. While the association of PSC with UC and its higher occurrence in men is extensively known, their study found that PSC was more common in both men and women, and both subtypes of IBD [32]. The changes in intestinal microbiome found in CD have also been implicated in the hepatobiliary inflammation that characterizes PSC. IBD and PBC share susceptibility genes as well. Although the evidence regarding the association of PBC with IBD is conflicting, a shared pathogenesis with CD involving Th17 response and granulomatous inflammation is evident [21,33]. AIH has been associated with IBD previously, both with and without concomitant PSC, carrying a 0.3% prevalence in UC patients [33].

### 4.5. Autoimmune Neurologic Disorders

Our study noted no association between IBD and autoimmune neurological disorders, including MS, ALS, or GBS. A genome-wide analysis has identified common genes between IBD and MS (IL2RA with CD, IL7R and FCGR2A with UC, and PTGER4 and STAT3 with both) [34]. There has been a previous association in case reports between IBD and MS [13,14]. Even some American studies reported an increased risk for MS in IBD patients [16]. A recent meta-analysis showed a significant association between MS and IBD as well, which remained true for both UC and CD and was stronger in women [35].However, results from our study do not indicate any such association and possibly warrant future research to explain the discrepancy. IBD has not been frequently associated with a higher risk of ALS, barring a Swedish study which found an association with UC [14]. There have even been reports of a negative association and possible protective effect of one over the other [36]. To our knowledge, GBS has not been documented with IBD, which is consistent with our study, which found no association. These were not associated with higher odds of in-hospital mortality.

### 4.6. Autoimmune Endocrine Disorders

An association between immune-mediated endocrine conditions and IBD has also been reported in the past. A significant association between IBD and Addison’s Disease, type 1 diabetes, hypothyroidism, and Graves’ disease was seen. Danish and Swedish studies have had similar findings with UC but have not found associations with CD [14,21]. Few previous studies have argued against this association, including a European meta-analysis, which did not find an overall increased risk for type 1 diabetes in IBD patients, but certain subgroups from different regions showed an association [37]. In our study, type 1 diabetes was associated with lower odds of having IBD. Elevated IL-18 levels and PTPN22 genes are shared by IBD and Type 1 DM [23]. While IL-18 likely influences Th1 response, PTPN22 is known to promote beta cell death in type 1 DM and intestinal barrier dysfunction in IBD. The earliest report of autoimmune thyroid disease in IBD dates back to 1962 [38]. While some studies report the considerable prevalence of autoimmune thyroid disease in IBD, the Manitoba study contrastingly found a lower prevalence of the same in IBD cases as compared to control. However, they found an increased risk of autoimmune thyroid disease specifically in patients of UC aged 40–59 years. Existing literature largely supports an association between Graves’ and UC [14]. A pediatric study found an association between hypothyroidism and IBD [15]. Documentation of Addison’s disease in IBD is scant, with the genotype-based meta-analysis reporting no linkage either.

### 4.7. Autoimmune Connective Tissue Disorders

Our study found significant associations between IBD and several connective tissue disorders. This included AS, BD, RA, Sarcoidosis, Sjogren Syndrome (SS), SLE, PR and DLE. AS is perhaps one of the most widely described diseases in association with IBD. Moreover, a concordance in the occurrence of AS in IBD has been observed in siblings [14]. Both AS and IBD are known to involve gene mutations in IL23 R and ERAP2 [23]. The presence of RA in IBD has been documented in European studies, and similar findings have been reported in children [15]. IBD shows a genetic overlap with RA. While PTPN22 and IL2RA are seen in both RA and CD, IL2and IL21 mutations are shared by RA and UC [23]. The Th1 and Th17 have been suggested to influence the development of RA, although the exact mechanism by which gut dysbiosis acts remains to be understood [39]. One study associated sarcoidosis with UC [21]. A link between sarcoidosis and CD is, however, easier to explain as both involve granulomatous inflammation mediated by Th1 and Th17. Moreover, common gene mutations in NOD2 and IL23 receptor have been noted in both [21]. SLE has been associated with UC [14]. Like RA, the overlap between IBD and SLE has also been observed in children [15]. SS and PR have been associated with IBD overall, while PR has also been restricted to UC in certain studies.

### 4.8. Autoimmune Vasculitis

Autoimmune vasculitis conditions, namely Takayasu arteritis, PAN, GCA, Wegners’ and GPA were also significantly associated with IBD in our study. A higher incidence of PAN in IBD has also been noted, more so in UC. GPA has been reported with CD and represents a diagnostic conundrum since the manifestations of both diseases show some overlap. An association between Takayasu arteritis and IBD is supported by previous studies, with CD and Takayasu sharing a similar pathogenesis characterized by granulomatous inflammation [40]. While Takayasu arteritis shows a stronger association with CD and females, GCA is more common in UC and males [41].

## 5. Limitations

This study has several limitations inherent to the use of large administrative databases such as the National Inpatient Sample. First, reliance on ICD-10-CM coding introduces a risk of misclassification bias, as diagnostic codes may not always reflect confirmed clinical diagnoses. Detection bias is also possible, as hospitalized patients with chronic conditions like IBD may undergo more extensive evaluations, increasing the likelihood of co-diagnoses such as autoimmune diseases. While adjustments were made for demographic variables and the Elixhauser comorbidity index, important confounders such as smoking status, environmental exposures, medication use (including immunosuppressants and biologics), genetic factors, and disease duration or severity are not captured in the NIS. Additionally, the cross-sectional design precludes any assessment of temporal relationships or causality between IBD and associated autoimmune conditions. The associations observed, particularly with in-hospital mortality, reflect risk during the index hospitalization and may not represent long-term outcomes. Stratification by IBD subtype (Crohn’s disease vs. ulcerative colitis) was not performed. Both conditions were included in the analysis using their respective ICD-10 codes; future studies may benefit from stratified analyses to identify disease-specific autoimmune associations.

## 6. Conclusions

The strongest associations were identified with primary sclerosing cholangitis, ankylosing spondylitis, autoimmune hepatitis, and celiac disease. Interestingly, IBD also demonstrated independent associations with several less commonly reported autoimmune conditions, including Addison’s disease, Behçet’s disease, and Takayasu arteritis. In contrast, a few autoimmune disorders, such as type 1 diabetes, showed no significant association or even an inverse relationship. Further, the study highlights the high burden of autoimmune comorbidities among hospitalized IBD patients and their association with in-hospital mortality. Specific autoimmune conditions such as polymyositis, AIHA, and thrombotic microangiopathy are linked to elevated mortality risk during hospitalization.

## Figures and Tables

**Table 1:** Patient demographics, stratified by the presence of IBD.

	Absence of IBD *n* (%)	Presence of IBD *n* (%)	*p*-Value
Age Categories			<0.001
18–44	39,180,126 (28.0)	518,360 (34.0)	
45–65	39,511,457 (28.2)	486,845 (32.0)	
>65	61,260,887 (44.0)	520,350 (34.0)	
Gender			<0.001
Male	59,448,781 (42.0)	665,530 (44.0)	
Female	80,503,690 (58.0)	860,025 (56.0)	
Race			<0.001
White	93,732,609 (66.8)	1,202,635 (78.8)	
African American	21,460,831 (15.3)	171,125 (11.2)	
Hispanic	15,778,430 (11.3)	93,165 (6.1)	
Asian/Pacific islander	3,930,613 (3.0)	18,390 (1.2)	
Native American	873,360 (0.6)	5,675 (0.4)	
Other	4,176,628 (3.0)	34,415 (2.3)	
Insurance			<0.001
Medicare	66,862,465 (49.3)	655,570 (44.2)	
Medicaid	25,763,816 (18.9)	224,660 (15.3)	
Private	37,174,100 (27.6)	546,910 (36.9)	
Uninsured	5,650,175 (4.2)	53,760 (3.6)	
Income			<0.001
Lowest quartile	42,924,734 (30.0)	375,405 (25.0)	
Second quartile	36,708,956 (26.0)	390,265 (26.0)	
Third quartile	32,967,139 (24.0)	392,715 (26.0)	
Highest quartile	27,351,642 (20.0)	367,170 (24.0)	
Elixhauser comorbidities			<0.001
0	20,353,951 (14.5)	183.270 (12)	
1	17,719,254 (12.7)	255,670 (16.8)	
2	20,065,959 (14.3)	269,820 (17.7)	
3 or more	81,813,307 (58.5)	816,795 (53.5)	

**Table 2:** Autoimmune comorbidities, stratified by the presence of IBD and the results of multivariate logistic regression analysis identifying the comorbidities between autoimmune conditions and IBD.

Autoimmune Skin Conditions	Absence of IBD n (%)	Presence of IBD n (%)	*p*-Value	aOR (95% CI)	*p*-Value
Bullous pemphigoid	47,220 (0.03)	435 (0.03)	0.14		
Pemphigus vulgaris	13,980 (0.01)	195 (0.01)	0.14		
Psoriasis	767,620 (0.5)	20,375 (1.3)	<0.001	2.35 (2.28–2.43)	<0.001
Autoimmune muscle disease					
Polymyositis	64,950 (0.05)	600 (0.04)	0.08		
Myasthenia gravis	207,225 (0.15)	2245 (0.1)	0.90		
Autoimmune hematological disease					
Autoimmune hemolytic anemias	71,930 (0.05)	990 (0.06)	0.003	1.30 (1.12–1.51)	0.001
Idiopathic thrombocytopenic purpura	276,325 (0.2)	3420 (0.2)	0.003	1.10 (1.02–1.20)	0.01
Pernicious anemia	76,735 (0.05)	1935 (0.1)	<0.001	2.47 (2.23–2.73)	<0.001
Autoimmune gastrointestinal and hepatic disorders					
Celiac disease	180,245 (0.1)	7150 (0.5)	<0.001	3.08 (2.92–3.26)	<0.001
Primary biliary cirrhosis	55,755 (0.04)	2335 (0.15)	<0.001	3.82 (3.47–4.21)	<0.001
Autoimmune hepatitis	100,885 (0.07)	4595 (0.3)	<0.001	4.01 (3.73–4.31)	<0.001
Primary sclerosing cholangitis	7435 (0.01)	7345 (4.8)	<0.001	80.26 (74.04–87.00)	<0.001
Autoimmune neurologic disorders					
Amyotrophic lateral sclerosis	70,360 (0.05)	600 (0.04)	0.09		
Multiple sclerosis	719,790 (0.5)	8670 (0.6)	0.0001	0.96 (0.91–1.01)	0.08
Guillain-Barre syndrome	90,780 (0.1)	1180 (0.1)	0.01	1.13 (0.99–1.29)	0.06
Autoimmune endocrine disorders					
Addison disease	123,200 (0.01)	4040 (0.3)	<0.001	2.68 (2.47–2.90)	<0.001
Graves’ Disease	186,100 (0.1)	2995 (0.2)	<0.001	1.40 (1.28–1.52)	<0.001
Type-1 diabetes mellitus	1,692,589 (1.2)	12,590 (1.0)	<0.001	0.52 (0.50–0.54)	<0.001
Autoimmune connective tissue disorder					
Ankylosing spondylitis	80,205 (0.06)	8050 (0.5)	<0.001	8.88 (8.42–9.38)	<0.001
Bechet’s disease	13,675 (0.01)	830 (0.05)	<0.001	4.29 (3.64–5.05)	<0.001
Rheumatoid arthritis	2,658,864 (2.0)	55,935 (4.0)	<0.001	2.07 (2.03–2.12)	<0.001
Sarcoidosis	384,720 (0.3)	4605 (0.3)	0.0067	1.12 (1.04–1.19)	0.002
Sjogren syndrome	256,890 (0.2)	4955 (0.3)	<0.001	1.64 (1.53–1.76)	<0.001
Systemic lupus erythematosus	836,735 (0.6)	15,650 (1.0)	<0.001	1.49 (1.43–1.55)	<0.001
Polymyalgia rheumatica	354,010 (0.3)	4545 (0.3)	<0.001	1.31 (1.22–1.40)	<0.001
Discoid lupus erythematosus	62,540 (0.04)	1110 (0.07)	<0.001	1.58 (1.38–1.80)	<0.001
Autoimmune vasculitis					
Takayasu arteritis	5645 (0.004)	215 (0.01)	<0.001	3.15 (2.28–4.34)	<0.001
Thrombotic microangiopathy	31,880 (0.02)	395 (0.03)	0.2651		
Polyarteritis nodosa	16,040 (0.01)	380 (0.02)	<0.001	2.13 (1.69–2.69)	<0.001
Giant cell arteritis	82,095 (0.06)	1080 (0.01)	0.0061	1.36 (1.19–1.56)	<0.001
Wegener’s granulomatosis	54,060 (0.04)	790 (0.05)	0.0007	1.32 (1.11–1.57)	0.001

**Table 3:** Demographic factors and autoimmune conditions associated with in-hospital mortality in patients with IBD.

Age Categories	Adjusted Odds Ratio	95% Confidence Interval	*p*-Value
18–44			
45–65	2.47	2.19–2.80	<0.001
>65	5.05	4.41–5.78	<0.001
Gender			
Male			
Female	0.85	0.81–0.90	<0.001
Insurance			
Medicare			
Medicaid	1.12	0.99–1.27	0.07
Private	1.05	0.96–1.15	0.30
Uninsured	1.20	0.96–1.51	0.12
Elixhauser comorbidities			
0			
1	3.17	1.90–5.27	<0.001
2	7.22	4.41–11.81	<0.001
3 or more	32.74	20.23–53	<0.001
Autoimmune comorbidities			
Psoriasis vulgaris	0.53	0.38–0.74	<0.001
Polymyositis	2.56	1.18–5.54	0.017
Autoimmune hemolytic anemia	2.60	1.32–5.12	0.006
Idiopathic thrombocytopenic purpura	2.19	1.54–3.10	<0.001
Amyotrophic lateral sclerosis	1.97	0.85–4.55	0.111
Hypothyroidism	0.70	0.64–0.76	<0.001
Systemic sclerosis	1.69	0.98–2.92	0.059
Polymyalgia rheumatica	0.72	0.47–1.09	0.116
Thrombotic microangiopathy	4.94	2.22–10.97	<0.001
Giant cell arteritis	1.37	0.71–2.66	0.346
Wegners granulomatosis	1.57	0.68–3.66	0.293

## Data Availability

The original contributions presented in this study are included in the article. Further inquiries can be directed to the corresponding author.
